# Superficial thrombophlebitis in the forearm leading to entrapment of the radial nerve branch: a first case report and literature review

**DOI:** 10.1186/s12891-024-07545-4

**Published:** 2024-06-01

**Authors:** Yinwei Zhang, Weijie Zhou, Jun Zhou, Weitao Chu, Jun Fan, Hui Lu

**Affiliations:** 1 Department of Orthopedics, Jinyun People’s Hospital, No.299 Ziwei Nouth Road, Lishui, Zhejiang Province 321400 People’s Republic of China; 2 Department of Orthopedics, No., 903 Hospital of PLA Joint Logistic Support Force, #40 Airport Road, Hangzhou, 31003 People’s Republic of China; 3 Department of Orthopedics, The First Affiliated Hospital, Zhejiang University, #79 Qingchun Road, Hangzhou, Zhejiang Province 31003 People’s Republic of China

**Keywords:** Superficial thrombophlebitis, Radial nerve entrapment

## Abstract

This article reports a case of a female patient admitted with swelling and subcutaneous mass in the right forearm, initially suspected to be multiple nerve fibroma. However, through preoperative imaging and surgery, the final diagnosis confirmed superficial thrombophlebitis. This condition resulted in entrapment of the radial nerve branch, leading to noticeable nerve entrapment and radiating pain. The surgery involved the excision of inflammatory tissue and thrombus, ligation of the cephalic vein, and complete release of the radial nerve branch. Postoperative pathology confirmed the presence of Superficial Thrombophlebitis. Through this case, we emphasize the importance of comprehensive utilization of clinical, imaging, and surgical interventions for more accurate diagnosis and treatment. This is the first clinical report of radial nerve branch entrapment due to superficial thrombophlebitis.

## Introduction

Superficial thrombophlebitis (ST) refers to an inflammatory condition affecting the superficial veins, often accompanied by the formation of blood clots [1]. It has long been believed that ST is a benign, self-limiting condition, which typically resolves rapidly on its own [2]. This clinical diagnosis is characterized by discomfort, erythema, induration, perivenous edema, and a reddish cord along the vein [3]. The lump resulting from superficial thrombophlebitis can be palpated along the course of the superficial branch of the radial nerve, leading to compression and subsequent development of Wartenberg syndrome [4]. Initially, Wartenberg syndrome was thought to be caused by an inflammation of the superficial radial sensory nerve, but gradually it was found to be caused by nerve entrapment [5]. Robert Wartenberg originally described the compression of the radial nerve's superficial branch in 1932, where is caused by entrapment of the superficial branch of the radial nerve at this site, where the nerve originates beneath the muscles [5, 6]. This paper presents the first reported case of superficial radial nerve branch compression caused by thrombophlebitis.

## Case description

**Fig. 1:**
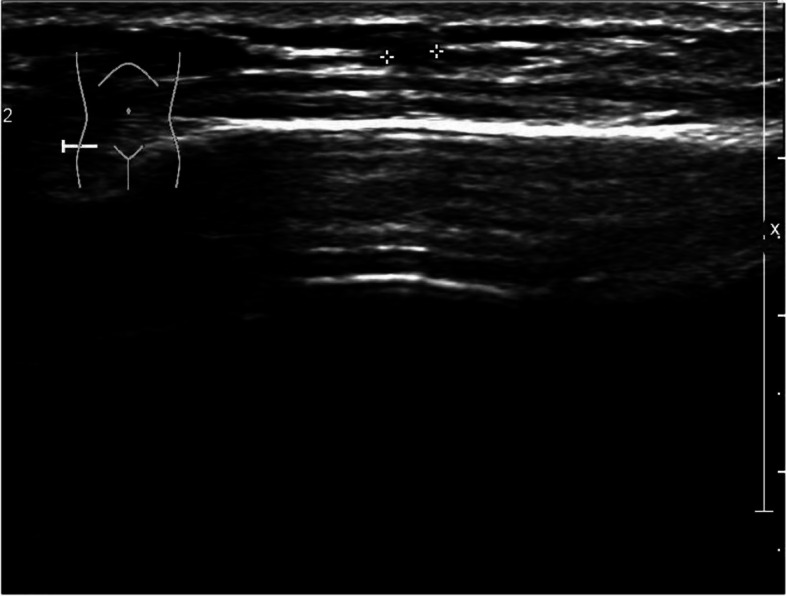
Superficial ultrasound reveals thrombus formation

**Fig. 2:**
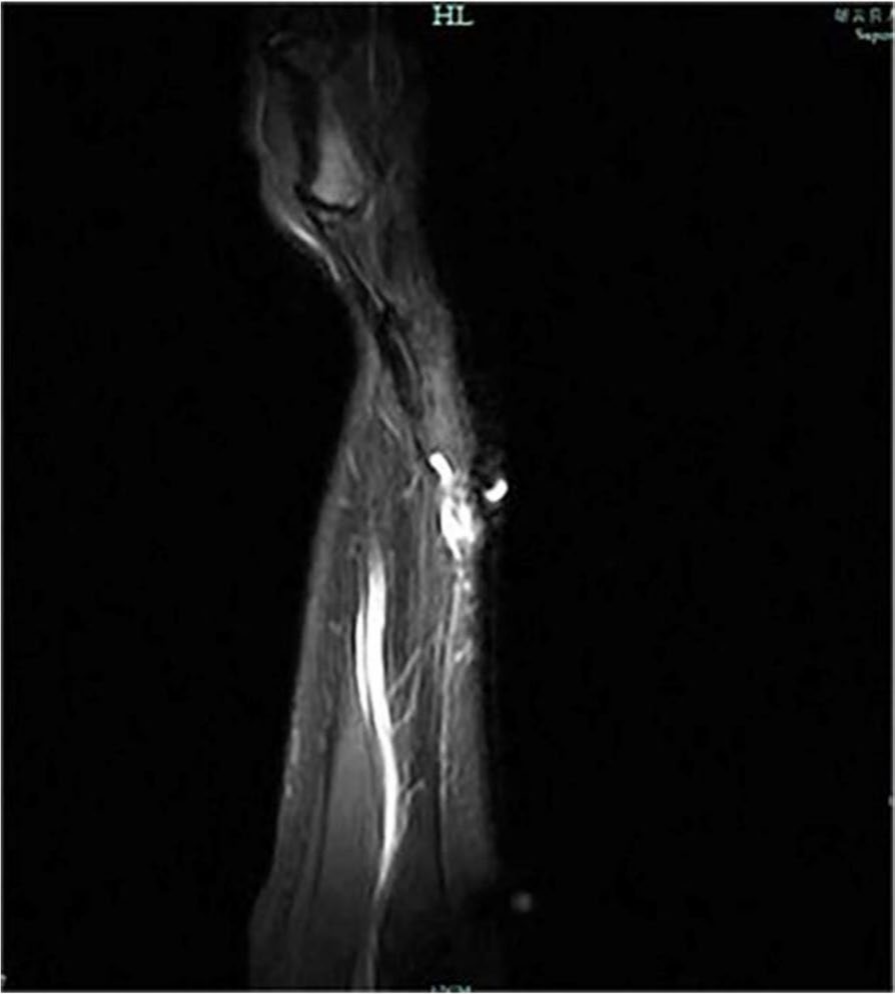
MRI shows inflammatory high signal

**Fig. 3:**
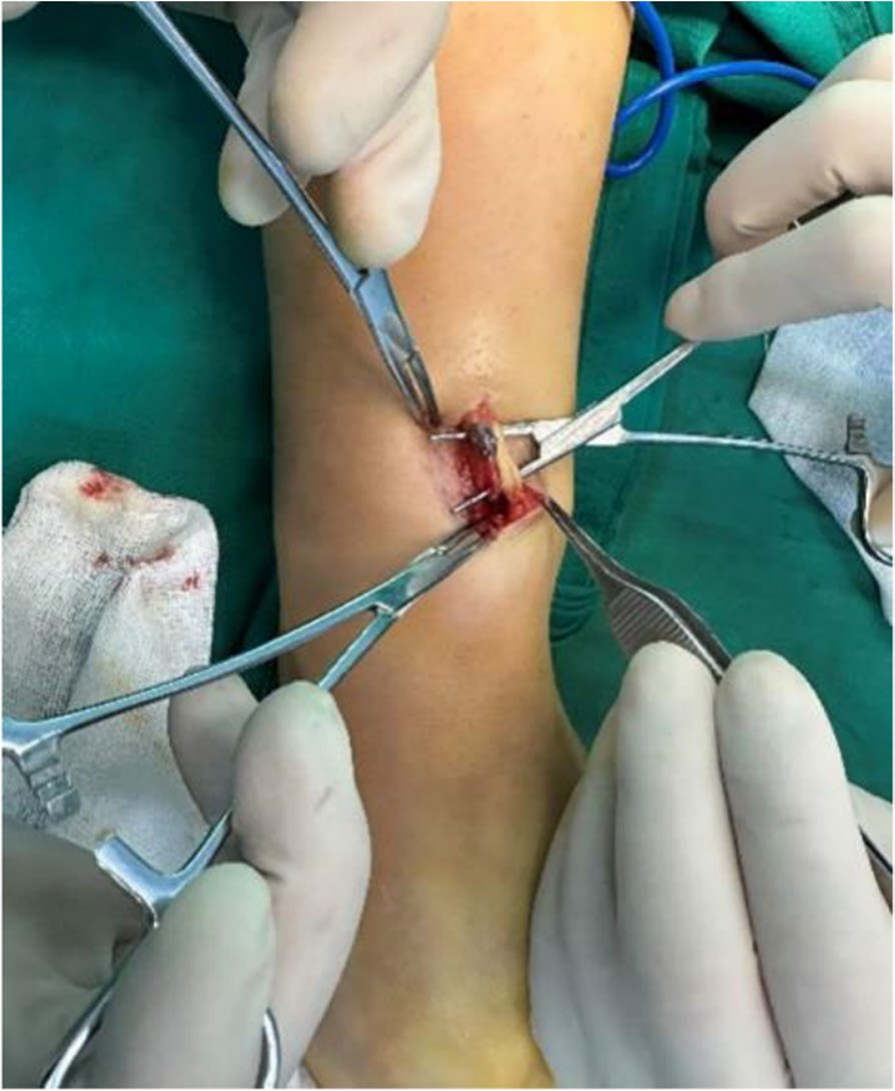
Intraoperative anatomy: Thrombosis of cephalic vein, and the superficial thrombophlebitis compress the superficial branch of radial nerve

**Fig. 4:**
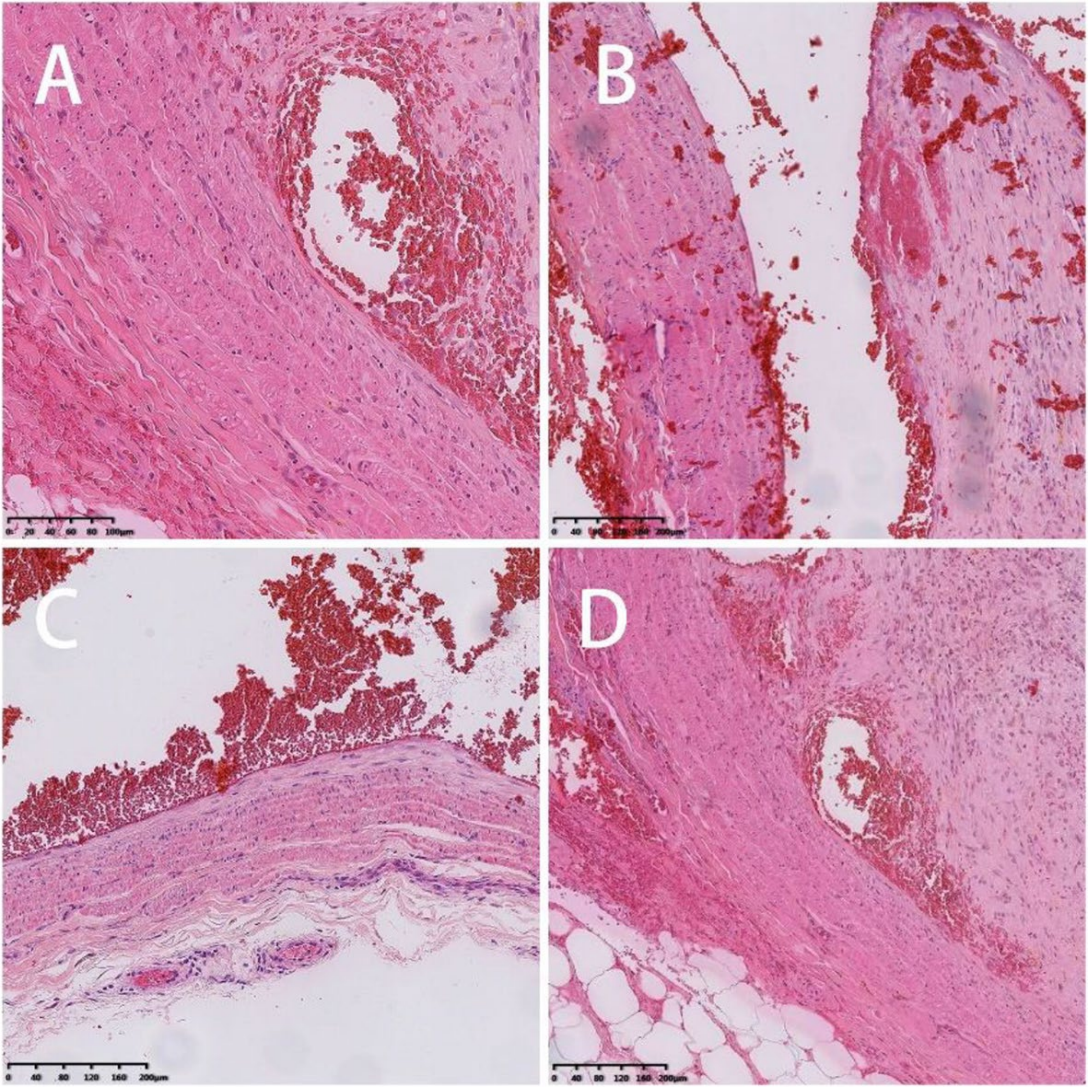
Postoperative pathological findings: vascular lumen fibrous tissue hyperplasia, phagocytosis of hemosiderin tissue cells aggregation, thrombosis. (**A**-**D**: H&E; **A**: × 20 magnification, **B**: × 10 magnification, **C**: × 10 magnification, **D**: × 10 magnification)

The patient presented with swelling on the dorsal aspect of the right forearm, accompanied by redness on the radial side. Subcutaneous nodules were palpable, and occasional numbness at the tiger's mouth was reported. The Tinel's sign is positive for the superficial branch of the radial nerve and radiating pain symptoms raised concerns about potential neurological disorders. Preoperative ultrasound indicated multiple hypoechoic masses interconnected within the subcutaneous muscle layer, with the largest measuring 7 × 3x3 mm, suggestive of multiple nerve fibromas (Fig. 1). Magnetic resonance imaging further revealed localized edema and inflammation on the radial side of the forearm with indistinct borders, strongly supporting the necessity for surgery (Fig. 2). During the operation, a location-based incision under the brachial plexus block revealed locally inflamed subcutaneous fat. Segmental thrombus in the cephalic vein and compression of the radial nerve branch were observed with local fat infiltration. Measures taken included excision of inflammatory tissue and thrombus, ligation of the cephalic vein, and thorough release of the radial nerve branch (Fig. 3). Postoperative pathology confirmed the presence of Superficial Thrombophlebitis, further validating the effectiveness of the surgery (Fig. 4). This provided a precise diagnosis and direction for the patient's treatment.

## Discussion

Superficial thrombophlebitis refers to an inflammatory condition affecting the veins located immediately beneath the skin's surface. Superficial thrombophlebitis shares a common etiology with other thrombotic disorders [7]. However, varicose veins remain the most clinically identifiable risk factor [8]. There is some evidence suggesting a relationship between ST and venous thromboembolism [9]. In this case the patient did not find any of the above triggers. In the patient's forearm, there were no visible varicose veins. There was no history of recent pregnancy, trauma, birth control pills, superficial vein injection or venous catheterization and surgery. Ultrasound is the preferred diagnostic modality, which can be utilized to exclude venous thromboembolism (VTE) and determine the extent of the disease [10]. This clinical diagnostic is characterized by discomfort, erythema, induration, and perivenous edema, reddish cord along the vein [11]. The current treatment methods are to relieve symptoms, limit the expansion of thrombosis and reduce the risk of pulmonary embolism [12]. The treatment of ST includes pressure socks, limb elevation, pain control, low molecular weight heparin and surgical intervention [13].

During the operation, there was no obvious inflammatory infiltration of the superficial branch of the radial nerve and the lump resulting from superficial thrombophlebitis palpated along the course of the superficial branch of the radial nerve, leading to compression and subsequent development of Wartenberg syndrome [4]. The similar pain can be from the overlap of the lateral antebrachial cutaneous nerve which occurs in 75% of people [14]. Superficial position of the superficial branch of the radial nerve, which is easy to be injured, including trauma, external compression (wrist watch, plaster model), internal compression (ganglion cyst, lipoma, abscess, bone spur) and iatrogenic injury [15]. The combination of MR or ultrasound imaging and electrical diagnosis research can realize more accurate initial diagnosis and timely treatment [16]. Although the prognosis of compression neuropathy depends on the degree of nerve injury, early surgical intervention is very important [17, 18]. In this case, the superficial branch of the radial nerve was compressed by a thrombotic superficial vein, so the right forearm radial nerve compression release and hemangioma resection were performed.

This case emphasizes the importance of comprehensive use of clinical symptoms and imaging examinations when facing unexplained forearm swelling and neurological symptoms. Preoperative assessment assists in more accurately determining the nature of the lesion, guiding the surgical approach. The success of the surgery underscores the critical role of surgical intervention in definitive diagnosis and treatment, especially in dealing with similar complex cases [19].

## Conclusion

Superficial thrombophlebitis resulting in entrapment of the radial nerve branch in the forearm is a rare yet noteworthy condition that we have recently discovered. Through this case, we highlight the importance of timely comprehensive diagnosis and surgical treatment, providing valuable insights for similar cases.

## Data Availability

No datasets were generated or analysed during the current study.
